# Expression of a Hydroxycinnamoyl-CoA Shikimate/Quinate Hydroxycinnamoyl Transferase 4 Gene from *Zoysia japonica* (*ZjHCT4*) Causes Excessive Elongation and Lignin Composition Changes in *Agrostis stolonifera*

**DOI:** 10.3390/ijms23169500

**Published:** 2022-08-22

**Authors:** Di Dong, Zhuoxiong Yang, Yuan Ma, Shuwen Li, Mengdi Wang, Yinruizhi Li, Zhuocheng Liu, Chenyan Jia, Liebao Han, Yuehui Chao

**Affiliations:** 1 School of Grassland Science, Beijing Forestry University, Beijing 100083, China; 2 School of Biological and Food Engineering, Suzhou University, Suzhou 234000, China; 3 Inner Mongolia M-Grass Ecology and Environment (Group) Co., Ltd., Hohhot 010000, China

**Keywords:** *ZjHCT4*, G-lignin, S-lignin, creeping bentgrass

## Abstract

Hydroxycinnamoyl-CoA shikimate/quinate hydroxycinnamoyl transferase (HCT) is considered to be an essential enzyme for regulating the biosynthesis and composition of lignin. To investigate the properties and function of *ZjHCT4*, the *ZjHCT4* gene was cloned from *Zoysia japonica* with a completed coding sequence of 1284-bp in length, encoding 428 amino acids. The *ZjHCT4* gene promoter has several methyl jasmonate (MeJA) response elements. According to analysis of expression patterns, it was up-regulated by MeJA, GA_3_ (Gibberellin), and SA (Salicylic acid), and down-regulated by ABA (Abscisic acid). Ectopic *ZjHCT4* expression in creeping bentgrass causes excessive plant elongation. In addition, the content of G-lingnin and H-lingnin fell in transgenic plants, whereas the level of S-lingnin increased, resulting in a considerable rise in the S/G unit ratio. Analysis of the expression levels of lignin-related genes revealed that the ectopic expression of *ZjHCT4* altered the expression levels of a number of genes involved in the lignin synthesis pathway. Simultaneously, MeJA, SA, GA_3_, IAA, BR (Brassinosteroid), and other hormones were dramatically enhanced in transgenic plants relative to control plants, whereas ABA concentration was significantly decreased. Expression of *ZjHCT4* impacted lignin composition and plant growth via altering the phenylpropionic acid metabolic pathway and hormone response, as revealed by transcriptome analysis. HCTs may influence plant lignin composition and plant development by altering hormone content. These findings contributed to a deeper comprehension of the lignin synthesis pathway and set the stage for further investigation and application of the *HCTs* gene.

## 1. Introduction

**Figure 1:**
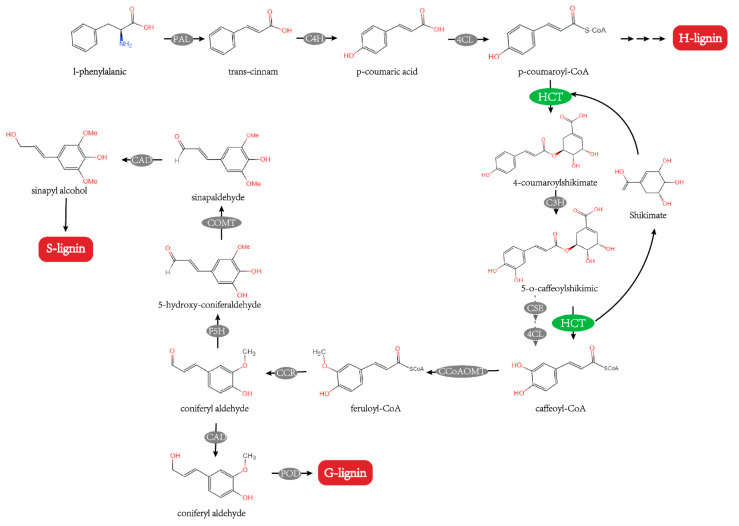
HCT participates in the lignin synthesis pathway [6,8,9]. PAL, phenylalanine ammonia-lyase; C4H, cinnamate 4-hydroxylase; 4CL, 4-hydroxycinnamate CoA ligase; C3H, coumarate 3-hydroxylase; CSE, caffeoyl shikimate esterase; CCoAOMT, caffeoyl-CoA O-methyltransferase; F5H, ferulate-5-hydroxylase; COMT, caffeicacid/5-hydroxy coniferaldehyde O-methyltransferase; CCR, cinnamoyl CoA reductase; CAD, cinnamyl alcohol dehydrogenase; POD, peroxidase.

Lignin, one of the essential components of the plant cell wall, serves vital biological tasks such as providing support for defense and transport [1,2]. It can increase the mechanical strength of plant tissues and stimulate the plant’s defensive mechanism [3]. Lignin is formed by the polymerization of interconnected lignin monomers, which can be categorized as p-hydroxyphenyl lignin (H-lignin), guaiacyl lignin (G-lignin), and syringyl lignin (S-lignin) based on the kinds of the monomers [4]. Through the phenylpropanoid pathway, one of the most significant branches of so-called plant specialized metabolism and lignin precursors are formed, and HCT is a crucial enzyme in the process’s initial steps [5]. HCT employs p-coumaroyl-CoA as the acyl donor and either shikimic acid or quinic acid as the acceptor, resulting in the respective shikimate or quinate ester [3,5]. Additionally, HCT exhibited reverse catalysis for caffeinyl COA. Caffeoyl-CoA, the precursor of guaiacyl and syringyl lignin units, is produced by the HCT transfer of the caffeoyl moiety of 5-O-caffeoylshikimic to CoA (Figure 1) [6]. HCT appears to serve a crucial role both upstream and downstream of the 3-hydroxylation stage in the phenylpropanoid pathway [7].

According to studies, the expression of HCTs continually modifies the level or composition of lignin and frequently has a significant effect on plant growth [10]. The *AtHCT* null mutation inhibited *Arabidopsis thaliana*’s growth [6]. Arabidopsis contains only one HCT, but other plants may possess numerous HCTs [11]. Studies conducted on pine trees (*Pinus radiata*) and alfalfa (*Medicago sativa*) indicate that the expression level of the *HCTs* gene can impact the synthesis and content of lignin in plants [9,12]. Downregulation of HCT in *Medicago sativa* decreases lignin levels and enhances bioethanol production by enhancing fodder quality and saccharification efficiency [10]. HCT-downregulated *Brachypodium distachyon* plants, which had a lodging phenotype, exhibited a considerable reduction in overall stem or leaf biomass and a fall in lignin content, although lignin composition was unaffected [11]. HCTs are BAHD acyltransferases, and they are capable of acylating a variety of substrates, demonstrating substrate variety [13,14,15,16]. The *PvHCT1a* and *PvHCT2a*, and *HCTs* genes in *Panicum virgatum* exhibited the predicted HCT activity [13]. Although *OsHCT4* in *Oryza sativa* demonstrates hydroxycinnamoyl transferase activity, the effect of *HCT4* on the lignin content of plants has not been investigated [17]. The ectopic expression of specific genes is one of the most effective methods to investigate the influence of gene expression and molecular design [18,19]. Ectopic expression of the *Betula platyphylla BpCCoAOMT* gene in *Nicotiana tabacum* enlarged reduced S-lignin content and interfered with the normal growth of plants [20].

*Zoysia japonica* is the most common warm-season turfgrass in the world. Due to its tolerance to drought and salt, it is frequently used on golf courses, sports fields, and in urban greening [21], whereas creeping bentgrass (*Agrostis stolonifera*) is an important cool-season grass species that is widely utilized as turfgrass on golf courses [22]. Lignin is a crucial component of turfgrass tissue’s mechanical strength and is directly associated with turf resistance [23].

In order to better understand the function of the *HCT*s gene and to lay the groundwork for future studies on its function in lignin synthesis and growth, the gene from *Zoysia japonica* was isolated, identified, and its ectopic expression and functional studies in *Agrostis stolonifera* were conducted. Exploring the mechanism by which the *HCTs* gene affects plant development and lignin composition is aided by research into the *ZjHCT4* gene.

## 2. Results

### 2.1. Identification and Bioinformatic Analysis of the ZjHCT4

**Figure 2:**
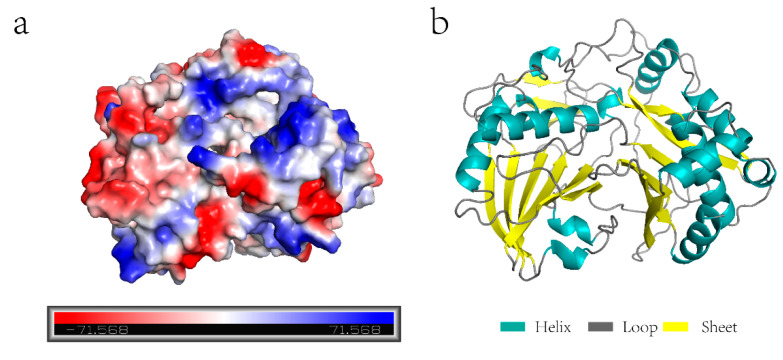
ZjHCT4 protein. (**a**) The electrostatic potential distribution of ZjHCT4. The value of electrostatic potential is proportional to the intensity of color, with blue indicating positive electrostatic potential and red representing negative electrostatic potential. (**b**) Simulation of the ZjHCT4 protein structure.

Using *Zoysia japonica* cDNA as a template, the full-length amplification of the hydroxycinnamoyl-CoA shikimate/quinate hydroxycinnamoyl transferase gene was identified, according to the Zoysia Genome Database. The full coding region of the *ZjHCT4* gene was 1284 base pairs long and encoded 428 amino acids. ZjHCT4 protein has the chemical formula C_2069_H_3205_N_551_O_606_S_20_, a molecular weight of 46.13 kD, and a predicted isoelectric point of 5.20. The protein’s structure was stable based on its 32.28 protein instability index value. Analysis of the ZjHCT4 protein’s three-dimensional structure revealed several positive and negative potentials on its surface (Figure 2a). The ZjHCT4 protein included the largest proportion of the loop, accounting for 42.06 percent of the total components (Figure 2b). The helix and sheet of the ZjHCT4 protein made up 36.21 percent and 17.99 percent, respectively. Phylogenetic tree analysis revealed that the HCT protein in *Zoysia japonica* was most closely linked to OsHCT4 in *Oryza sativa* (Supplementary Figure S1).

### 2.2. Upstream Sequence Analysis of ZjHCT4

**Figure 3:**
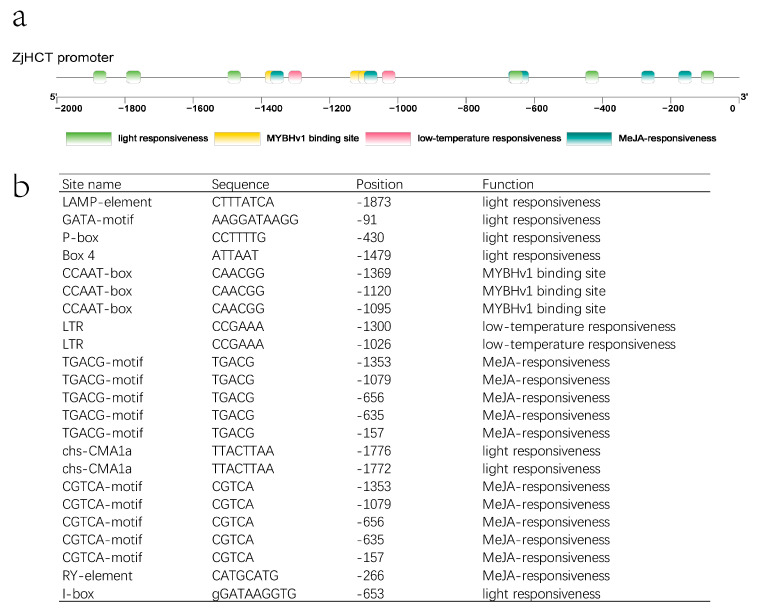
Analysis of the upstream sequence of ZjHCT4. (**a**) Cis-acting elements upstream of ZjHCT4. (**b**) A list of putative transcription factor binding sites upstream of ZjHCT4.

The upstream sequence was used to explore the cis-regulatory elements of the ZjHCT4 promotor. Several light responsiveness elements, such as LAMP-element (CTTTATC), GATA-motif (AAGGATAAGG), P-box (CCTTTTG), Box 4 (ATTAAT), chs-CMA1a (TTACTTAA), I-box (GGATAAGGTG), are found in the upstream region of ZjHCT4. The TGACG/CGTCA-motif (TGACG/CGTCA) and RY-element (CATGCATG) are identified as elements of the pathways of MeJA. In addition, low-temperature responsiveness elements LTR (CCGAAA) and MYBHv1 binding site CCAAT-box (CAACGG) are also found (Figure 3).

### 2.3. Subcellular Localization

**Figure 4:**
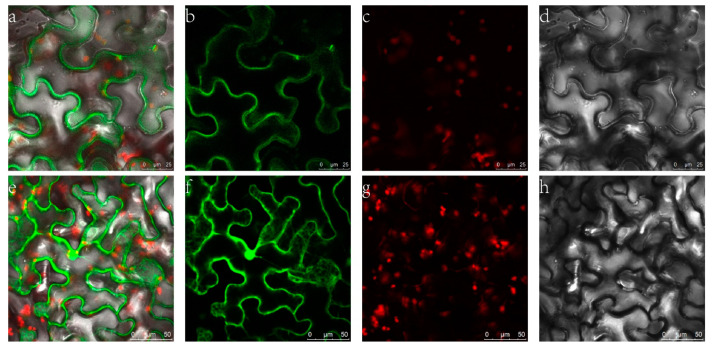
Subcellular localization of ZjHCT4. (**a**–**d**) Fusion protein of ZjHCT4 and YFP fluorescent detection. (**e**–**h**) YFP fluorescent detection. (**a**,**e**): merged signal; (**b**,**f**): FP fluorescence; (**c**,**g**): Y chlorophyll autofluorescence; (**d**,**h**): Bright light.

To confirm the cellular location of ZjHCT4, *Agrobacterium tumefaciens* was utilized to infect tobacco leaves with 35S::ZjHCT4: YFP. Transient expression of YFP and ZjHCT4 fusion proteins resulted in cytoplasmic fluorescence, whereas untargeted YFP was found throughout the cell. Analysis of subcellular localization revealed that ZjHCT4 is mostly cytoplasmic (Figure 4).

### 2.4. Ectopic Expression of ZjHCT4 in Creeping Bentgrass

**Figure 5:**
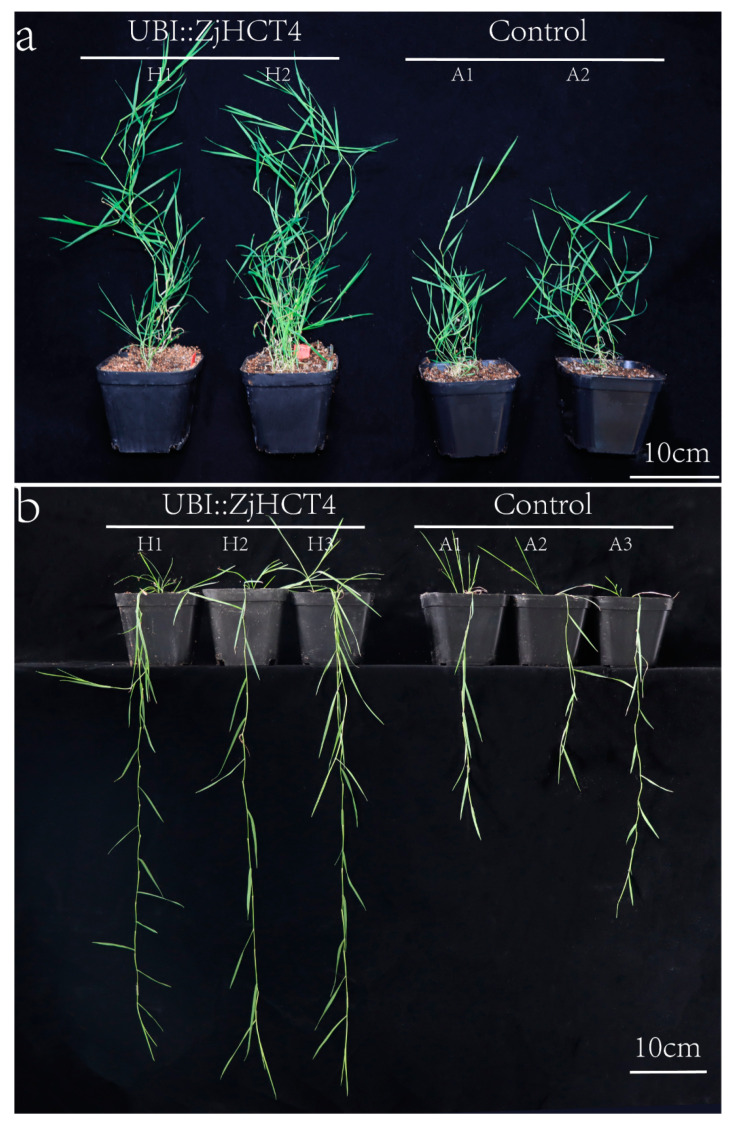
Excessive elongation phenotype of transgenic creeping bentgrass. (**a**,**b**) Comparison of the phenotypes of UBI::HCT transgenic creeping bentgrass and control at 30 days.

The agrobacterium-mediated method was utilized to obtain the transgenic UBI::ZjHCT4 *Agrostis stolonifera* lines (H1, H2, and H3), and the control plants lines (A1, A2, and A3) with the empty vector. The leaves of transgenic plants exhibited an exaggerated elongation phenotype in comparison to those of the control plants. After 30 days of development, the length of transgenic plants was 2.02 times greater than that of the control group (Supplementary Figure S2). These findings imply that ectopic production of *ZjHCT4* may accelerate plant development and result in excessive plant elongation (Figure 5).

### 2.5. Determination of Monomer Content of Lignin

**Figure 6:**
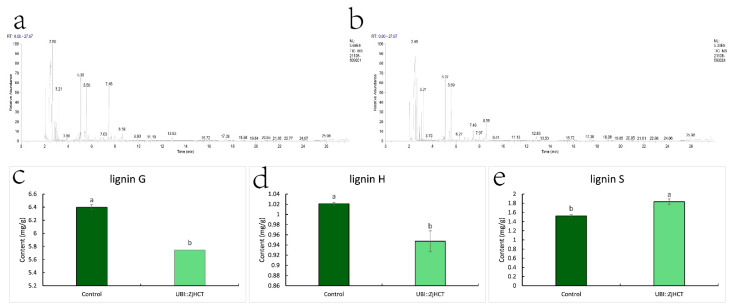
Change of monomer content of lignin. (**a**,**b**) Typical gas chromatograms show thioacidolysis products of transgenic (**a**) and control plants (**b**). (**c**–**e**) Monomer content of lignin G (**c**), lignin H (**d**), lignin S (**e**). Statistical significance of differences was assessed using the Student’s *t*-test. Different letters above the columns indicate significant differences (*p* < 0.05, n = 3).

Degradation of lignin polymers by thioacidolysis, measurement of lignin monomer degradation products, and disclosure of lignin monomer composition, as depicted in Figure 6. G units and H units were dramatically reduced after ectopic expression of *ZjHCT4*, falling to 0.90 and 0.93 times that of control plants. In contrast, the S units of the transgenic plants were 1.20 times greater than those of the control plants. From 0.24 to 0.32, the S/G ratio of genetically modified plants grew dramatically. These results demonstrate that ectopic expression of *ZjHCT4* has an influence on all three lignin monomers, with the greatest effect on S lignin.

### 2.6. Ecpotic Expression of ZjHCT4 Causes Hormonal Changes

**Figure 7:**
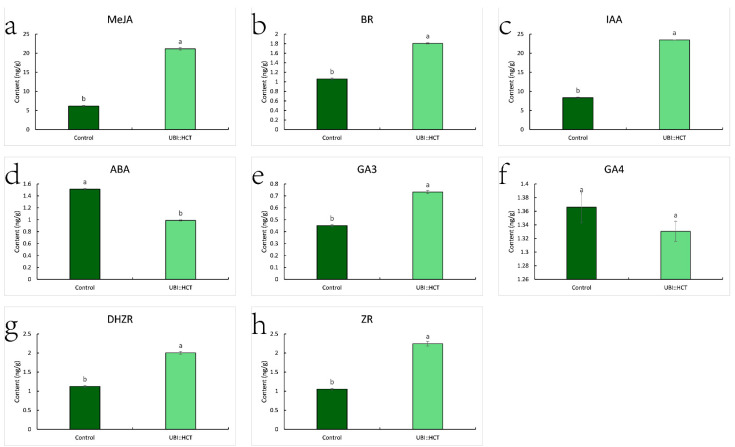
Hormone contents of transgenic and control plants, including MeJA (**a**), BR (**b**), IAA (**c**), ABA (**d**), GA_3_ (**e**), GA_4_ (**f**), DHZR (**g**), ZR (**h**). Statistical significance of differences was assessed using the Student’s *t*-test. Significant differences (*p* < 0.05, n = 3) are shown by different letters.

MeJA, BR, IAA, GA_3_, DHZR (Dihydrozeatinriboside), and Zr (Zeatin riboside) concentrations in transgenic plants were substantially greater than in control plants (Figure 7). Notably, IAA and MeJA levels in transgenic plants were 2.82 and 3.47 times higher than in control plants, respectively. In contrast, the levels of the hormones BR, DHZR, GA_3_, and ZR were 1.70, 1.78, 1.62, and 2.13 times those of the control group. In contrast, the ABA content of transgenic plants was drastically reduced to 62.27 percent of the control level. Despite the fact that the GA_3_ concentration of transgenic plants was 1.63 times that of the control group, there was no significant difference in GA_4_ content.

### 2.7. Expression Pattern of ZjHCT4 and the Lignin-Related Genes

**Figure 8:**
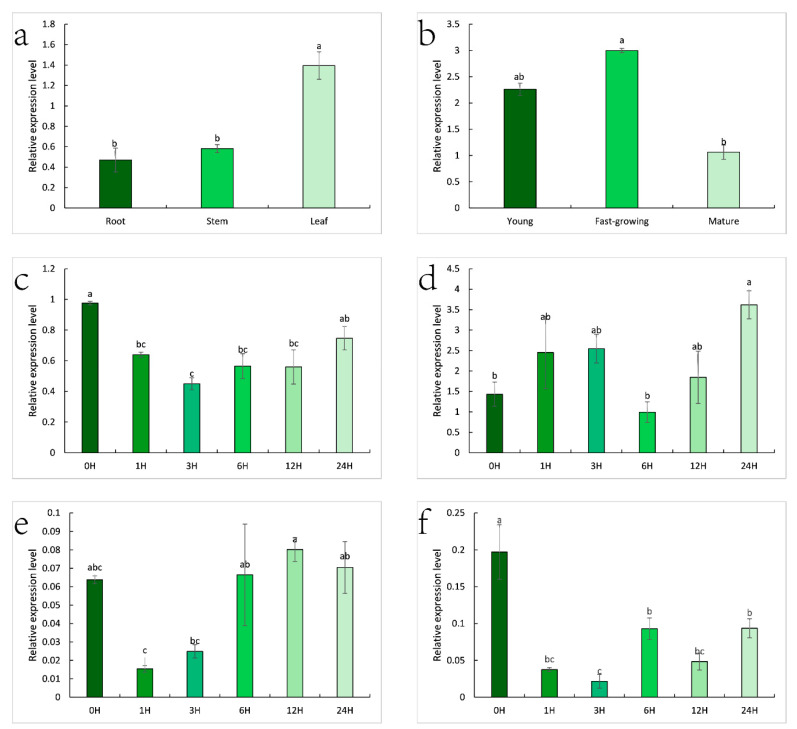
Expression pattern of *ZjHCT4* in *Zoysia japonica*. (**a**) Expression profiles of *ZjHCT4* expression in the root, stem, and leaf. (**b**) The pattern of *ZjHCT4* expression in leaves at various stages of development. (**c**–**f**) Leaf expression pattern after treatments with 0.5 mM SA (**c**), 10 mM ABA (**d**), 10 μM GA3 (**e**), and 10 mM MeJA (**f**). Statistical significance of differences was assessed using one-way ANOVA with Duncan test. Significant differences (*p* < 0.05, n = 3) are represented by various letters above the columns.

Analysis of expression patterns revealed that the *ZjHCT4* gene is expressed differently in distinct *Zoysia japonica* tissues. The expression level of the *ZjHCT4* gene was 2.97 and 2.40 times higher in leaves than in roots and stems, respectively (Figure 8a). The level of gene expression in mature leaves was significantly greater than in mature leaves, and the levels of gene expression in young and fast-growing leaves were 2.13 and 2.82 times greater than in senile leaves, respectively (Figure 8b).

SA, GA_3_, and MeJA all inhibited the expression of the *ZjHCT4* gene (Figure 8c,e,f). The expression of the *ZjHCT4* gene was initially reduced after treatment with SA and GA3, but subsequently gradually restored. After 24 h of therapy, the levels of expression recovered to untreated levels. After 1 h and 3 h of MeJA treatment, the expression level of *ZjHCT4* dropped to 0.19 times that of untreated samples and 0.11 times that of untreated samples, respectively. Even though the expression of *ZjHCT4* increased over time, after 24 h, the expression was only 0.47 times that of untreated *ZjHCT4*.

Within 12 h of ABA treatment, the expression level of *ZjHCT4* did not change significantly. However, after 24 h, the expression level increased to 2.53 times that without ABA. This indicates that ABA may have a minor stimulatory effect on *ZjHCT4* expression (Figure 8d).

**Figure 9:**
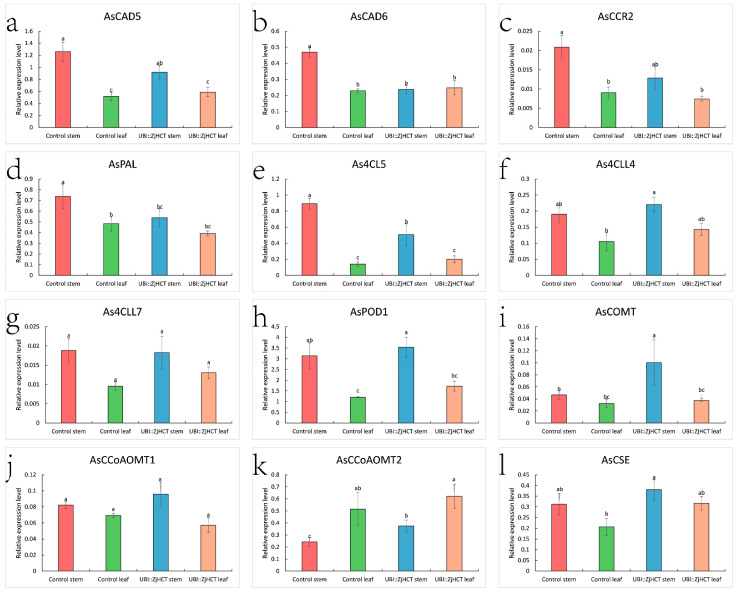
Expression levels of *AsCAD5* (**a**), *AsCAD6* (**b**), *AsCCR2* (**c**), *AsPAL* (**d**), *As4CL5* (**e**), *As4CLL4* (**f**), *As4CLL7* (**g**), *AsPOD1*(**h**), *AsCOMT* (**i**), *AsCCoAOMT1* (**j**), *AsCCoAOMT2* (**k**), *AsCSE* (**l**), in leaves and stems of control and transgenic plants. The stem of control (red), leaf of control (green), stem of transgenic plant (blue), and leaf of transgenic plant (pink) columns were separately compared using one-way ANOVA with Duncan test. Differences that are statistically significant (*p* < 0.05, n = 3) are denoted by different letters above the columns.

To understand the role of *ZjHCT4* in lignin synthesis pathway, the expression profiles of genes involved in lignin synthesis, *AsCAD5* (*cinnamyl alcohol dehydrogenase 5*), *AsCAD6* (*cinnamyl alcohol dehydrogenase 6*), *AsCCR2* (*cinnamoyl CoA reductase 2*), *AsPAL* (*phenylalanine ammonia-lyase*), *As4CL5* (*4-hydroxycinnamate CoA ligase 5*), *As4CLL4* (*4-hydroxycinnamate CoA ligase-like 4)*, *As4CLL7* (*4-hydroxycinnamate CoA ligase-like 7*), *AsPOD* (*peroxidase*), *AsCOMT* (*caffeicacid/5-hydroxy coniferaldehyde O-methyltransferase*), *CCoAOMT1* (*caffeoyl-CoA O-methyltransferase 1*), *CCoAOMT2* (*caffeoyl-CoA O-methyltransferase 2*), and *AsCSE* (*caffeoyl shikimate esterase*) were investigated in the leaf and stem of transgenic and control plants (Figure 9). Expression levels of *AsCAD5, AsCCR2, As4CLL4*, *As4CLL7*, *AsPOD1*, *AsCCoAOMT1*, and *AsCSE* did not differ significantly between transgenic and control plants. However, significantly decreased *AsCAD6, AsPAL,* and *As4CL5* were expressed in the stems of transgenic plants compared to control plants. The levels of *AsCOMT* and *AsCCoAOMTA2* expression in stems increased considerably. Although the expression levels of *As4CLL4*, *AsPOD1*, and *AsCCoAOMTA2*, and *AsCSE* in the leaves of transgenic plants were higher than those of control plants, they were not substantially different.

### 2.8. Analysis of Differentially Expressed Genes

**Figure 10:**
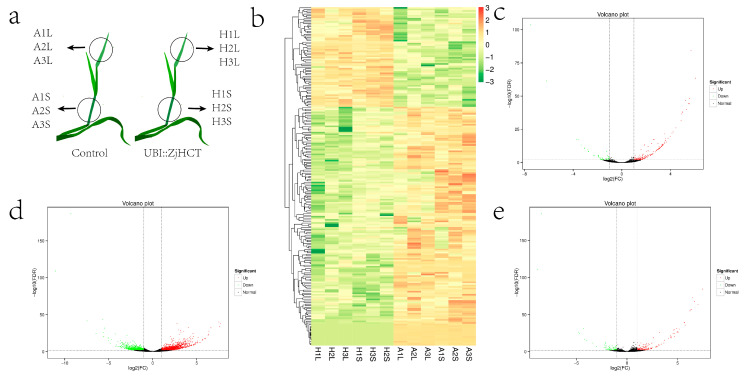
Transcriptome and differential expression gene analysis of transgenic vs. control plants. (**a**) Diagrammatic representation of transcriptome sampling. (**b**) Cluster diagram of expression patterns of differentially expressed genes. (**c**–**e**) Volcano plots of DEGs in leaves (**c**), stem (**d**), and aboveground parts (including leaves and stem, (**e**) of transgenic vs. control plants. DEGs were considered significantly different and are depicted by red dots (upregulation) and green dots (downregulation), at a corrected *q*-value ≤ 0.005.

Transgenic and control plants were chosen for sampling and transcriptome analysis (Figure 10a). On the DEGs (differentially expressed genes) that were screened, hierarchical cluster analysis was done, and genes with similar expression characteristics were clustered to illustrate the patterns. Clustering results of DEGs revealed that gene expression of transgenic plants and control plants differed significantly (Figure 10b). In leaves of transgenic vs. control plants, 210 DEGs were identified in the DEG analysis, with 163 upregulated genes and 447 downregulated genes (Figure 10c). The stems of transgenic vs. control plants contained a total of 2204 differently expressed genes, of which 1455 were up-regulated and 749 were down-regulated. The aboveground sections (leaves and stem) of transgenic vs. control plants had a total of 217 differentially expressed genes, with 153 up-regulated and 64 down-regulated. These results imply that ectopic expression of *ZjHCT4* may result in differential gene expression in stems and leaves, with stems containing a greater number of genes with differential expression.

**Figure 11:**
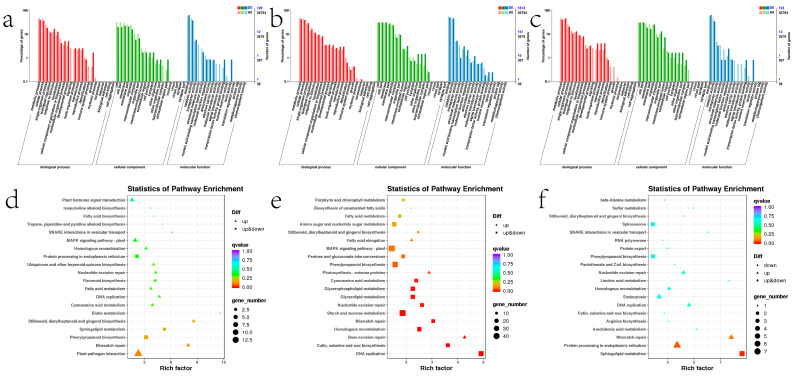
The GO enrichment and KEGG enrichment results of the up-regulated and down-regulated DEGs. GO enrichment results of DEGs in leaves (**a**), stems (**b**), aboveground parts (**c**), and KEGG enrichment results of leaves (**d**), stems (**e**), and aboveground parts (**f**) of transgenic and control plants with *q*-value ≤ 0.05 were considered significantly enriched.

To gain a better understanding of the general function of the DEGs identified, GO enrichment analysis and KEGG pathway enrichment analysis were performed utilizing the up-regulated and down-regulated DEGs, respectively, to gain a better understanding of the general function (Figure 11). It is notable that 59 and 7 differentially expressed genes are enriched in the “developmental process” and “growth” in stem, whereas 6 and 2 genes are enriched in leaf, respectively. This shows that the ectopic expression of *ZjHCT4* may have an effect on the genes involved in plant development and, consequently, on the plant growth process.

**Figure 12:**
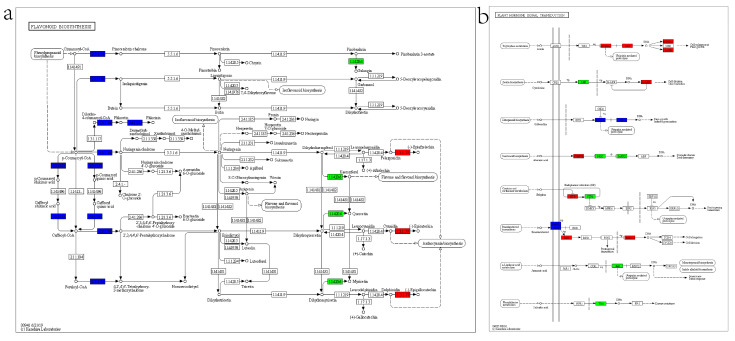
KEGG pathways for DEGs in transgenic and control stems. (**a**) DEGs mapped to flavonoid biosynthesis. EC:2.3.1.133, HCT; EC:2.3.1.74, chalcone synthase; EC:2.4.1.357, phlorizin synthase; EC:1.14.20.6, flavonol synthase; EC:1.3.1.77, anthocyanidin reductase. (**b**) DEGs involved in plant hormone signal transduction. AUX/IAA, auxin-responsive protein IAA; ARF, auxin response factor; SAUR, SAUR family protein; AHP, histidine-containing phosphotransfer peotein; ARR-A, two-component response regulator ARR-A family; DELLA, DELLA protein; TF, phytochrome-interacting factor 3; PYR/PYL, abscisic acid receptor PYR/PYL family; PP2C, protein phosphatase 2C; SNRK2, serine/threonine-protein kinase SRK2; ETR, ethylene receptor; CTR1, serine/threonine-protein kinase CTR1; BAK1, brassinosteroid insensitive 1-associated receptor kinase 1; BSK, BR-signaling kinase; BZR1/2, brassinosteroid resistant 1/2; JAZ, jasmonate ZIM domain-containing protein; TGA, transcription factor TGA.

The most prevalent KEGG pathways in stems are “Starch and sucrose metabolism”, “MAPK signaling pathway -plant”, and “Phenylpropanoid biosynthesis”. In addition, 11 and 39 differentially expressed genes in stems were localized to “Flavonoid biosynthesis” and “Plant hormone signal transduction”, respectively, whereas in leaves there were 2 and 4 gene localization (Figure 12 and Supplementary Figure S3).

The altered expression of multiple genes in the flavonoid biosynthesis pathway suggests that *ZjHCT4* may disrupt flavonoid biosynthesis and modify the synthesis of lignin precursors (Figure 12a and Supplementary Figure S3a). Meanwhile, ectopic expression of *ZjHCT4* can affect multiple genes in “Plant hormone signal transduction” pathway, so affect the signal transduction of auxin, cytokinine, gibberellin, abscisic acid, jasmonic acid, and salicylic acid (Figure 12b and Supplementary Figure S3b).

## 3. Discussion

The *ZjHCT4* gene represents a pivotal step for regulating the biosynthesis and composition of lignin. This study cloned the *ZjHCT4* gene from *Zoysia japonica*, which had a 1284-bp open reading frame encoding 428 amino acids.

HCT belongs to clade V of the BAHD acyltransferase superfamily, which includes enzymes that utilize coenzyme A-activated acyl donors and chemically varied acceptors such as organic acids, amines, or fatty acids [24]. ZjHCT4 is localized in the cytoplasm, which may be attributable to the absence of transporter peptides and sequences targeted to other organelles in the BAHD family of proteins. Additionally, previous research has demonstrated that BAHD family proteins are typically located in the cytoplasm [25].

Analysis of expression patterns revealed that the *ZjHCT4* gene is expressed differently in several tissues and growth stages of *Zoysia japonica*. The highest level of *ZjHCT4* expression was found in leaves, whereas the level of *ZjHCT4* expression in the fast-growing stage was much greater than that in the mature stage. This shows that *ZjHCT4* may play a role in plant development. The expression of *ZjHCT4* may be marginally stimulated by the hormone ABA. Previous research indicated that ABA greatly enhanced *CsHCT* expression levels in *Camellia sinensis*, which may be associated with HCT’s role in abiotic stress and the ABA signaling pathway [25]. Furthermore, it may be inhibited by the hormones SA, MeJA, and GA_3_, with MeJA having the highest inhibitory effect. Five TGACG/CGTCA motif elements and one RY element are found at 1353,1079,656,635,157 bp and 266 bp in front of the *ZjHCT4* CDS region, respectively, in the promoter of the *ZjHCT4* gene. These findings indicate that *ZjHCT4* may have a role in the control of MeJA hormone.

**Figure 13:**
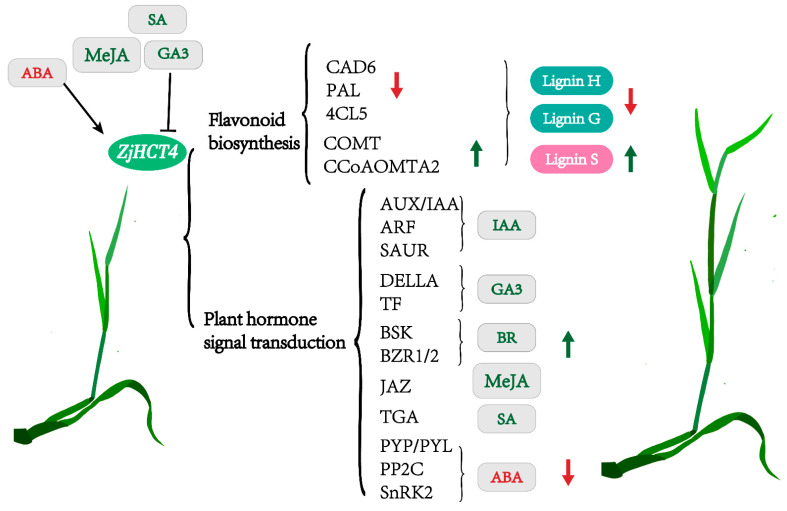
Model of *ZjHCT4* influences lignin composition and hormone content, resulting in an elongation pattern in *Agrostis stolonifera*. Red arrows indicate down, while green arrows indicate up.

In the meantime, transcriptome analysis demonstrated that heterotopic expression of *ZjHCT4* can influence the signal transduction of auxin, cytokinine, gibberellin, abscisic acid, jasmonic acid, and salicylic acid (Figure 13). Finally, results in the hormone content differential between transgenic and control plants are achieved. The MeJA concentration of ectopic *ZjHCT4*-expressing plants was 3.43 times greater than that of control plants. In the examination of the *ZjHCT4* expression pattern, the expression level of *ZjHCT4* reduced following the application of MeJA. These experimental results suggested that MeJA may negatively influence the expression of the *ZjHCT4* gene. The ectopic expression of *ZjHCT4* in the stem greatly elevated JAZ (Jasmonate ZIM domain-containing protein). It has been discovered that JAZ proteins are essential transcriptional repressors in jasmonate-regulated regulation. Stem and leaf JAZ are modified differently by the expression of *ZjHCT4*, which is remarkable (Figure 11 and Supplementary Figure S3). *JAZ* (C76945.graph c0; C82862.graph c0) was up-regulated in stem relative to the control, but down-regulated in the leaf. This may be a result of the different functions of the JAZ [26]. The overelongated phenotype of *Agrostis stolonifera* expressing *ZjHCT4* may result from its effect on hormone responsiveness and signal transduction (Figure 13).

Ectopic expression of lignin-related genes can be utilized to investigate the function and influence of these genes in lignin synthesis [18,19,20]. Changes in lignin content may influence the anatomical structure of stem vascular tissue and plant growth, resulting in a variation in biomass [27]. The ectopic expression of *ZjHCT4* alters the lignin composition of plants. GC/MS results revealed that both G and H lignin decreased in comparison to control plants. However, the proportion of S-type lignin increased significantly, suggesting its significance in lignin monomer transition. Lignin levels may affect development by influencing the formation of vessel walls, which may affect the solute transport [28,29]. In addition, investigations have revealed that it may influence pathway intermediates or the Mediator complex to effect plant development [30,31]. After ectopic expression of *ZjHCT4*, alterations in flavonoid production pathway gene expression were also observed (Figure 9, Figure 12 and Figure 13).

## 4. Materials and Methods

### 4.1. Plant Materials and Growth Conditions

Plants of *Zoysia japonica* (cv. ‘Compadre’), *creeping bentgrass* (cv. ‘Penn A-4’), *Arabidopsis thaliana,* and *Nicotiana benthamiana* were seeded in pots in a growth chamber (26/20 °C, 50% humidity, 16 h/8 h photoperiod).

### 4.2. Cloning of ZjHCT4 and Vector Construction

Total RNA was extracted from healthy zoysia of a 3-month-old plant. RNA isolation and cDNA synthesis followed previous descriptions [32]. The ORF sequence of *ZjHCT4* was amplified with primers listed in Supplemental Table S1. Coding sequences of *ZjHCT4* were recombined into the 3302YUBI vector (UBI::ZjHCT4) which were then transformed into *Agrobacterium tumefaciens* strain ‘EHA105’.

### 4.3. Generation of Transgenic Plants

Agrobacterium-mediated genetic transformation of *creeping bentgrass* was conducted following a protocol described [33,34]. The transgenic plantlets were identified on selective agar plates with glufosinate ammonium, and transferred to the controlled greenhouse conditions and subsequent experiments were conducted. Transgenic plants carrying 3302YUBI were used as a negative control.

### 4.4. Subcellular Localization Analysis

*ZjHCT4* were cloned into 3302Y vector to generate a 35S::ZjHCT4:YFP vector. *A. tumefaciens* strain EHA105 was transformed using 35S::ZjHCT4:YFP and transiently expressed in *Nicotiana benthamiana* leaves. A Leica TCS SP 8 confocal microscope was used to capture the YFP fluorescence signal.

### 4.5. Bioinformatics Analysis

BLAST homology identification was used to retrieve HCT proteins (Supplementary Table S1) from the NCBI database for phylogenetic tree analysis. The neighbor-joining (NJ) method of MEGA 6.0 was utilized for phylogenetic tree analysis. The PlantCARE database was utilized for the examination of cis-regulatory elements (http://bioinformatics.psb.ugent.be/webtools/plantcare/html (accessed on 18 November 2021)), as well as TBtools [35,36]. Phyre v2.0 was used to investigate the tertiary structure of the proteins (www.sbg.bio.ic.ac.uk/phyre2/ (accessed on 5 May 2021)) and version 2.5.2 of PyMOL.

### 4.6. Expression Levels of ZjHCT4 Gene and Lignin-Related Genes

The total RNA from several tissues (roots, stems, and leaves) and three developmental stages (young, fast-growing, mature) of leaves were isolated to examine the *ZjHCT4* expression pattern. In addition, we treated 3-month-old *Zoysia japonica* with 10 μmol/L ABA, 10 μmol/L MeJA, 200 μmol/L ETH, 300 mmol/L NaCl, and 20% PEG4000, respectively. The tissues were collected after being induced for 0, 1, 3, 6, 12, and 24 h. The beta-actin gene from *Zoysia japonica* (GenBank accession number: GU290546) was chosen as the internal reference gene [32]. The expression patterns of the *AsCAD*, *AsCAD6*, *AsCCR2*, *AsPA*, *As4CL*, *As4CLL4*, *As4CLL7*, *AsPOD1*, *AsCOMT*, *AsCCoAOMT*, *AsCCoAOMT*, and *AsCSE* in transgenic and control plants were evaluated using RT-PCR. The actin gene (GenBank accession number: DY543529) was used as an internal control [37]. For each treatment, three biological duplicates were established. The primer sequences used above are presented in Supplementary Table S2. The relative expression levels were calculated using the 2^−ΔΔCt^ method [38].

### 4.7. Hormone Contents Assay

The control and transgenic plants’ one-month-old leaves were collected and crushed with liquid nitrogen after rapid freezing. After analysis by icELISA (indirect competitive enzyme-linked immunosorbent assay), the OD value of each sample at 490 nm were measured on a Multiskan FC microplate photometer (Thermo Scientific, Waltham, MA, USA). The calculation method is as described above [39].

### 4.8. Transcriptomic Analysis of DEGs

The leaves and stems of 30-day-old transgenic and control plants were examined using the RNA-Seq technique. Library building, sequencing, differential expression gene screening, and transcriptome analysis were carried out as described previously. BMKCloud (www.biocloud.net, (accessed on 20 November 2021)) was utilized for the analysis of hierarchical clustering, GO, and KEGG.

### 4.9. Determination of Lignin Composition

Lignin monomer was determined using thioacidolysis and the Raney nickel desulfurization technique. As its trimethylsilyl derivatives, lignin-derived monomers and dimers were identified by gas chromatography-mass spectrometry (GC/MS) and quantified by gas chromatography (GC) [40].

## 5. Conclusions

The ZjHCT4 is essential for lignin synthesis and monomer formation. MeJA has the strongest inhibitory effect of the four hormones ABA, SA, GA_3_, and MeJA. The ectopic expression of *ZjHCT4* in *Agrostis stolonifera* resulted in a phenotype of excessive elongation, and the content of MeJA dramatically increased in comparison to the control plants. Moreover, the expression levels of multiple genes in the lignin biosynthesis pathway were altered in transgenic plants. In transgenic plants, the amount of G-lignin and H-lignin reduced whereas the amount of S-lignin grew, resulting in an increase in the S/G ratio. Transgenic study further revealed that exogenous expression of *ZjHCT4* altered gene expression in the flavonoid biosynthesis and the plant hormone signal transduction pathway.

## Data Availability

The data presented in this study are available on request from the corresponding author.

## Supplementary Materials

The supporting information can be downloaded at: https://www.mdpi.com/article/10.3390/ijms23169500/s1.
