# Supplementary material for: Expression of a Hydroxycinnamoyl-CoA Shikimate/Quinate Hydroxycinnamoyl Transferase 4 Gene from Zoysia japonica (ZjHCT4) Causes Excessive Elongation and Lignin Composition Changes in Agrostis stolonifera
Source: Int J Mol Sci. 2022 Aug 22;23(16):9500. doi: 10.3390/ijms23169500 (PMC9408870; doi:10.3390/ijms23169500)
Supplement: Supplementary file 1 [file ijms-23-09500-s001.zip › ijms-1831877-supplementary.pdf]

Expression of a Hydroxycinnamoyl-CoA Shikimate/Quinate Hydroxycinnamoyl Transferase 4 Gene from *Zoysia japonica* (*ZjHCT4*) Causes Excessive Elongation and Lignin Composition Changes in *Agrostis stolonifera*

Di Dong [1,†], Zhuoxiong Yang [1,†], Yuan Ma [1], Shuwen Li [1], Mengdi Wang [1], Yinruizhi Li [1], Zhuocheng Liu [1], Chenyan Jia [2*], Liebao Han [1,*] and Yuehui Chao [1,*]

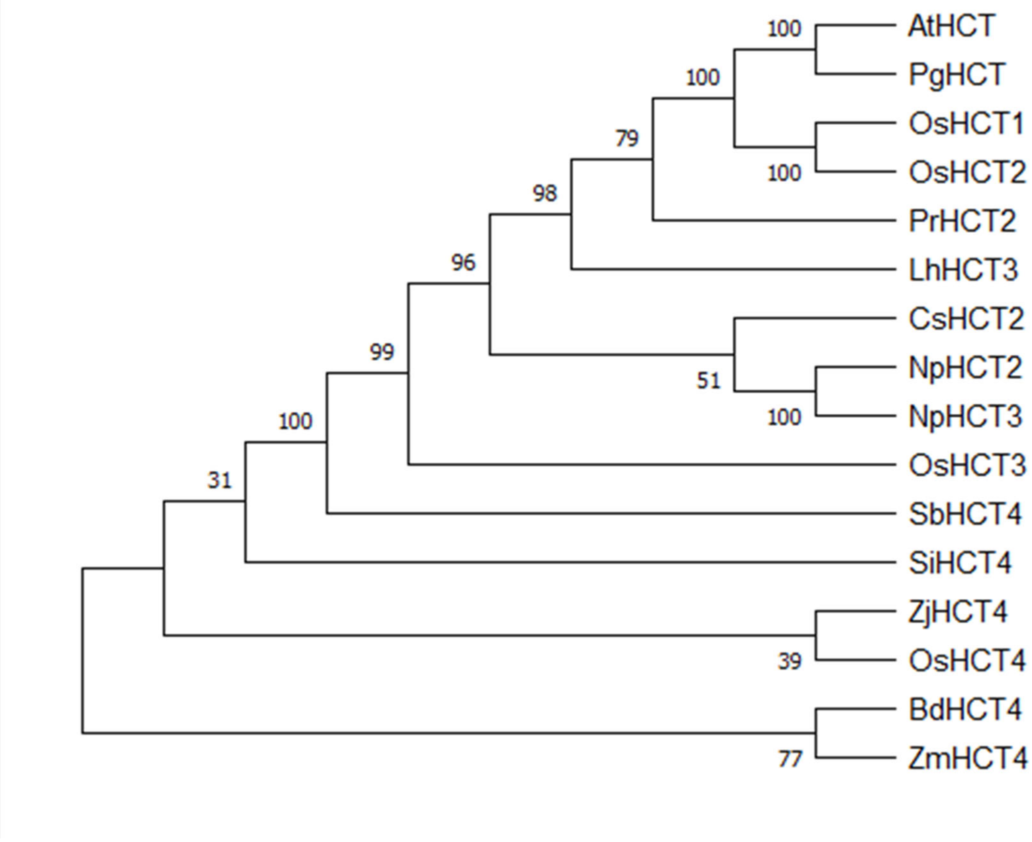

Supplementary Figure S1. Analysis of HCTs proteins. Protein sequence accession numbers are as follows: AT5G48930.1, AtHCT, *Arabidopsis thaliana*; AZS54120.1, PgHCT, *Punica granatum*; Q0JBZ8.1, OSHCT1, *Oryza sativa*; Q6K638.1, OsHCT2, *Oryza sativa*; ASM47232.1, PrHCT2, *Parasponia rugosa*; AKN80440.1, LhHCT3, *Lonicera hypoglauca*; KAF4871374.1, CsHCT2, *Colletotrichum siamense*; AXU39903.1, NpHCT2, *Narcissus papyraceus*; AXU39904.1, NpHCT3, *Narcissus papyraceus*; Q5SMM8.1, OsHCT3, *Oryza sativa*; XP_002462397.1, SbHCT4, *Sorghum bicolor*; XP_004964652.1, SiHCT4, *Setaria italica*; Q5SMM6.1, OsHCT4, *Oryza sativa*; XP_010229918.1, BdHCT4, B*rachypodium distachyon*; PWZ06330.1 ZmHCT4, *Zea mays*.

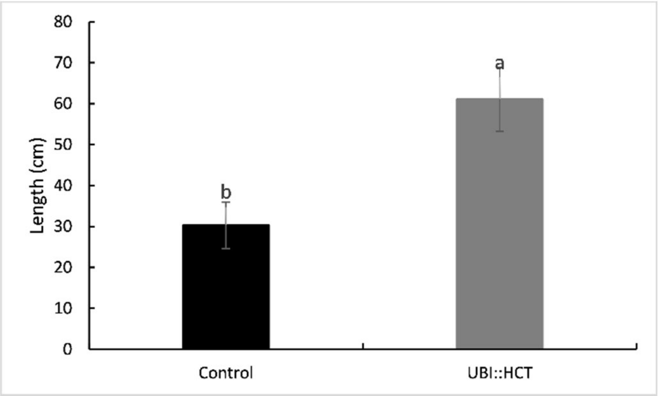

Supplementary Figure S2. Length comparison of transgenic plants and control plants after 30 days of growth. Statistical significance of differences was assessed using the Student's t-test. Different letters above the columns indicate significant differences ($p < 0.05$, $n = 10$).

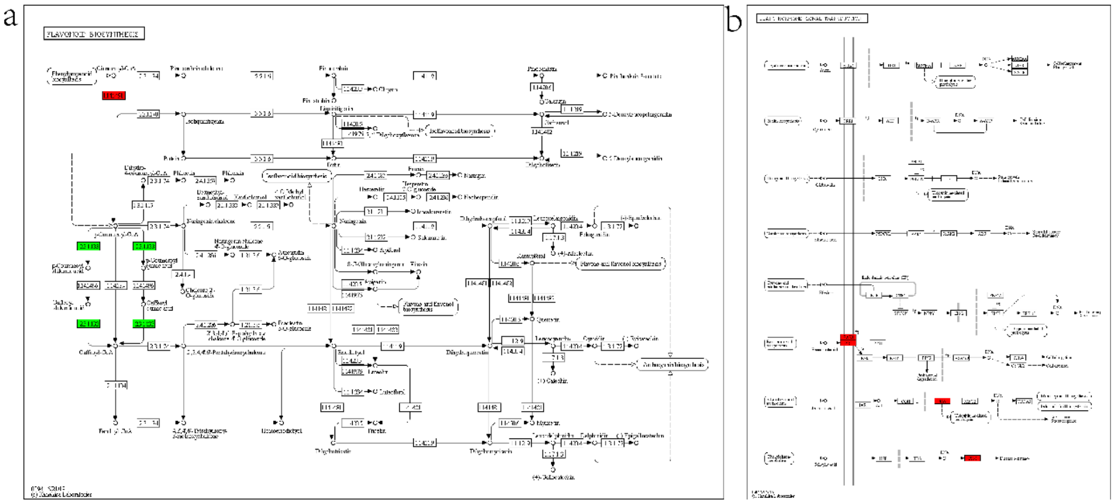

Supplementary Figure S3. KEGG pathways for DEGs in transgenic and control leaves. (a) DEGs mapped to flavonoid biosynthesis. EC:2.3.1.133, HCT; EC:1.14.14.91, trans-cinnamate 4-monooxygenase. (b) DEGs involved in plant hormone signal transduction

Supplementary Table S1. Protein sequence of HCT proteins.

Protein sequence
>ZjHCT4<br>MATVVTSVVVATAGAVWSNDAARRGYTTVYYRNGAADAVKDSSKAVAYAGRGVDTGRVVDCTGG<br>AVVTARSDYVDDNVCMRDVATANCAVVTYRCGGVVGAMHHSVVDARGAAHTWASISRGDTAHA<br>AVCDHSAARTTVYDHRYKVDVTASTYASAIITMTKGVSAKARCAGASTRSVVAVWCVCRARAATRY<br>SMIDMRTRDAGYGNAVIRTSVSATVGVVANVGYAARRAAATTGDYTRSVDYGVDAMNRSGISRAH<br>RAISWVGMSYKADGWGAAMGAIMYYSGVYVMNAAGKDGDAVSSMRKVADASVA
>SiHCT4<br>MAVVEVLTSEVAVPAEETPAGAIWLSNLDLAARRGYTPTVYFFRSNGEPGFFAAEVVKESLARALVAFY<br>PLAGRLGVDATTGRVQIDCNGEGAVFVTARSGRYALDDLMSEFAPCREMRDLFVPPTPPPNPPCPLL<br>FVQVTRLRCGSVVLGQAMHHSACDARGAAHFFETWASIARGDAAAAPVPPCFDHGLLAARPERAV<br>TYDHPEYMPEPEPVDAAAASEYASAIITMTKAQVAALRARCPGASTFRAVVALVWRCACRARSLPHD<br>AETRLYSMIDMRARLDPPLPPGYFGNAVVRTSVSATAAEVVSSPVGHVARRALAATSQGGDYARSLV<br>DYLEGVDAMNLPRSGISRAHLRAISWVGMSLYKADFGWGAPAFMGPALMYYSGFVYVMNAAGKD<br>GDLALVLSLEPESMPEFRKVFAEELARLDVV
>BdHCT4<br>MAKVEVLATELVVPAGETPGGSIWLSNLDLAARRGYTPTVYFYRPKNHGAVDPEEAFFAAGAVKCSL<br>AKALVAFYPLAGRLGLDDAAGRLQIDCTGEGAVLVTARSDHYALDELMSEFVPCGEMRDLFVPATPA<br>PNPPCALLLAQVTRLRCGGVVLGLALHHSVVDARSAAHFVETWASISRGNHQDAPLVPPCFDHRLL<br>DARPYPARAVLYDHPEYKPEPAPVDVTPVSASTYASAIITVSKKQVAALRARCAGASTFRAVVALVWQ<br>CACRARALAPGAETRLYSMIDMRPRLAPPLPQGYFGNAVVRTSAVVTVDEVVSSPVAYGARRARAA<br>TSQGDDYARSLVDYLETVDTMNLPRSGISRAHLRAISWMGMSLSDADFGWGAPAFMGPALMYYSG<br>FVYVMNAPGKDGAVALALSLEPDSMPEFKKTFADELARLEVEV
>ZmHCT4<br>MAMVELLSTELVVPAEETPAGAVWLSNLDLAARRGYTPTVYFYRTNGKPEFFETDAVKDSLARALVSF<br>YPLAGRLGLDAATGRVQIDCTGEGAVFVTARWEQYALDELVGEFVPCDEMRALLVPATPAPNPPCPL<br>LFAQVTRLRCGGVVLGLALHHSVVDARSAAHFVETWASIARGGAGAGAGTPLPPCFDHRLLNARPP<br>GARAVAYDHPEYKAEAPAAADGGAGGGAAAGYASAIVTLSKAQVAALKARCAGASTFRAVVALVW<br>QCACRARALPGDAETRLFSMVDMRARLAPPLPPGYFGNAVVRTSALATAGEVTGNPVGYAARRALA<br>ATSPGDDYARSLVDYLEGVDAMNLPRSGISRAHLRAISWMGMSLHDSDFGWGAPVFMGPALMYYS<br>GFVYVMQAPGKEGAVALALSLEPESMPEFRKVFAQELARLQAI
>SbHCT4<br>MAVEVVTSELVAPSETTPRRALWLSNLDLAARNGYTPTVYFFRRRCPQPQDGSGGDDDDRAPQQPS<br>PDFFSADVLRAALAAALVQFYPFAGRLRAGRDDDGRAEIDCNAAGALFVVARSAAALEDFDGFAPSK<br>AMNDTFVPKYDSTAGPDAPLLLLQVTFFRCGGVTLGTAMHPFVIDGRSAFHFIRTWASIARGDTAAA<br>AVPPSLDRTPLRARPLPTVLFDHTYEYGGRTSRPPTTGGNNKAAEYASAILRVTGAQAAALRARAGAV<br>STFRALVAHVWRCACAARALAPDAESRLYTMVDMRARLSPPLPDAFFGNAVARTSVSAVVGDLLAN<br>PLGFGARRLRAATGHGDEYARSLLDYLETADLAALPRGGLAGTDLRVISWLGMPSYDADFGWGEPAL<br>LAPALMYYPGFVYLLNCPGNGKGGGVAVAVALEPERMERFKELFFEELAALE
>AtHCT<br>MKINIRDSTMVRPATETPITNLWNSNVDLVIPRFHTPSVYFYRPTGASNFFDPQVMKEALSKALVPFYP<br>MAGRLKRDDDGRIEIDCNGAGVLFVVADTPSVIDDFGDFAPTLNLRQLIPEVDHSAGIHSFPLLVLQV<br>TFFKCGGASLGVGMQHHAADGFSGLHFINTWSDMARGLDLTIPPFIDRTLLRARDPPQPAFHHVEY<br>QPAPSMKIPLDPSKSGPENTTVSIFKLTRDQLVALKAKSKEDGNTVSYSSYEMLAGHVWRSVGKARGL<br>PNDQETKLYIATDGRSRLRPQLPPGYFGNVIFTATPLAVAGDLLSKPTWYAAGQIHDFLVRMDDNYL<br>RSALDYLEMQPDLSALVRGAHTYKCPNLGITSWVRLPIYDADFGWGRPIFMGPGGIPYEGLSFVLPSP<br>TNDGSLSVAIALQSEHMKLFEKFLFEI

>PgHCT<br>MIIAVKESTMVRPASETPSYALWNSNVDLVVPSMHTPSVYFYRPTGASDFFDPTVLKSALSRALVPFYP<br>MAGRLKRDEDGRIEIDCNGEGVLFVVAETTSKVDDFGDFAPTLELRKLIPAVDYSAGITSYPVLVLQVTY<br>FKCGGASLGVGMQHHVADGFSGLHFVNSWSDIARGLDVSVPPFIDRTLLRARDPPQPQFEHIEYQPP<br>PALKAPPSQAAHKPGTEPTTVSIFRMTKEQLGTLKGKSQDGNTVQYSSYEMLAGHVWRCACKARGL<br>PDDQETKLYIATDGRARLHPPLPPGYFGNVIFTATPLAVAGDLQSKPTWYAASRIHDALVRMDNDYL<br>RSALDYLELQPDLSALVRGAHTFRCPNLGITSWVRLPIHDADFGWGRPIFMGPGGIAYEGLAFVLPSP<br>DNDRSLSVAISLQTEHMKVFEKLLYEI
>OsHCT1<br>MAITVRRSTMVRPAWETPRVRLWNSNLDLVVPRFHTPSVYFYRRGPEGGGAPEGFFDGERMRRALA<br>EALVPFYPMAGRLARDEDGRVEIDCNGEGVLFVEADAPDASVDDYGDFAPTMELKRLIPAVDYTDDI<br>SSFSLLVLQVTYFKCGGVSLGVGMQHHVADGMSGLHFINSWSDLCRGTQIAIMPFIDRTLLRARDPP<br>TPSYPHVEYQPAPAMLSSVPQSVTANKTTPPPTAVDIFKLTRSDLGRLRSQLPSGEGAPRFSTYAVLAA<br>HVWRCVSLARGLPSEQPTKLYCATDGRQRLQPPLPEGYFGNVIFTATPLAEAGKVTSGLADGAAVIQE<br>ALDRMNDSYCRSALDYLELQPDLSALVRGAHTFRCPNLGLTSWVRLPIHDADFGWGRPVFMGPGGI<br>AYEGLAFVLPSANKDGSLSIAISLQAEHMEKFRKLIFEV
>OsHCT2<br>MKINVRGSTMVRPAEETPRVRLWNSSLDLVVPRFHTPSVYFFRRGEAAAAEGGSYFDGERMRRALAE<br>ALVPFYPMAGRLAHDEDGRVEIDCNGEGVLFVEADAPGATVDDFGDFAPTMDLKRLIPTVDYTDGIS<br>SFPILVLQVTHFKCGGVALGVGMQHHVADGFSGLHFINSWADLCRGVPIAVMPFIDRTLVRARDPPA<br>PSHPHVEYQPAPAMLAPEPPQALTAKPAPPPTAVDIFKLSRSDLGRLRSQLPRGEGAPRYSTYAVLAA<br>HVWRCASLARGLPAEQPTKLYCATDGRQRLQPSLPDGYFGNVIFTATPLAEAGRVTGSLADGAATIQ<br>SALDRMDSGYCRSALDYLELQPDLSALVRGAHTFRCPNLGLTSWVRLPIHDADFGWGRPVFMGPGG<br>IAYEGLAFVLPSASGDGSLSVAISLQAEHMEKFRKMIFDF
>PrHCT2<br>MAKIKVKEMTMIKPAAKTHRRTVWLSGLDLMNSDVHTPFVYFYKPNGASDFFNPAVLKEALIKVLVSF<br>YPMAGRFSLDSSRRLEIECNDEGALFVVAESSSVIDDFGDFTPTPDMRKLVPAVDYSGGISSYPFLVLQ<br>VTYFKCGGVSLGVGVEHRVVDGPAAFHFVNEWSNIARGLKLAIPPVFDRTIFCSRNPPQVVFNHFEY<br>QLTPGTKTSQQSSTTSTNSQSGVTTPTVSVFEITIEQLNILKAKAKEDSNIVNYSTYEILAGHFWKCASIA<br>RAIPNSQETRLHFAANGRNLRMKPRPQPGYFGNAVFVATSAVVASDLQYKPLWYAASRIRETLVRM<br>DDNYLRSAIDYLELFPNTKRDGAHFYESPNFRVTSWMKMPIYEANFGWGQPLYVGPAAMKHEGKAL<br>IIPTANKDGSLLLILVLQHEQMEVFKKLFYEYYYNDVKQVRYSLRAKL
>LhHCT3<br>MVNLIESCVIKPAKHTPNGLMSLSEFDQVGAITHASIVYLYQSPIDFDSAIVTLKDSLSNVLVTFYPLAG<br>RLHDIGKGRFEVDCNAIGAQFLVAESESRIEDFGDFHPTPQLQTLVPQVDTKAPVHEQPLLLVQVTKL<br>GCGGMCLGLAISQIMVDGSSGFHFTNEWARLARGEQLKNPPFLDRRVLQAKEPALLEPSFDHPEYGP<br>PPVLMGHTDALEEIKKETTIVMLKLDKKDIEKLKNKANNGENNYPLCYSRYEVVAGHMWRCACKARR<br>HASQQLTWLYFPMDVRNRIEPPLPQFYFGNAVYRAAATSTSGELISKPLSYASSKIREAKAKATDEYLR<br>SSMMFLKNLPDVSQCRNFHSPIPEAFYGNPNIDINNSWGRQPSDGLDFGWGEEIYGGTAAHGSEGK<br>SIIMRGSHEDGSFLIMLHLQVAHIEDFKKFFYGDI

>LhHCT3<br>MVNLIESCVIKPAKHTPNGLMSLSEFDQVGAITHASIVYLYQSPIDFDSAIVTLKDSLSNVLVTFYPLAG<br>RLHDIGKGRFEVDCNAIGAQFLVAESESRIEDFGDFHPTPQLQTLVPQVDTKAPVHEQPLLLVQVTKL<br>GCGGMCLGLAISQIMVDGSSGFHFTNEWARLARGEQLKNPPFLDRRVLQAKEPALLEPSFDHPEYGP<br>PPVLMGHTDALEEIKKETTIVMLKLDKKDIEKLKNKANNGENNYPLCYSRYEVVAGHMWRCACKARR<br>HASQQLTWLYFPMDVRNRIEPPLPQFYFGNAVYRAAATSTSGELISKPLSYASSKIREAKAKATDEYLR<br>SSMMFLKNLPDVSQCRNFHSPIPEAFYGNPNIDINNSWGRQPSDGLDFGWGEEIYGGTAAHGSEGK<br>SIIMRGSHEDGSFLIMLHLQVAHIEDFKKFFYGDI
>CsHCT2<br>MSRVTRDVQTHWVRPCQPQGSSGDQVWPISDFDYIQPAINIPLVAIYALPSDANGPSVIKDLRVGLA<br>ETLGQYRQFGGRLQINDETGQHNIKTTDNDVVELSVQYHDGPDDDYVPSYAALEKDAFPPMLLDSK<br>KLLPRIMTERQNLTQHGDFHEDDAPVTMFMVTFIRGGLVIGVGLHHLATDVSGLDGFLESWAANTK<br>RLVAGLPLAPFDHSIMDRALLTHSGPAPDVKRWTELDQRLKTVKLLDTAPQPPPPEFKMPATSEVLFH<br>FPRSKINQLKANASRTEPNTWISSYDAIMALCWRCVSRARQSSVTGDTATTLMFAVNGRGRLKPPLS<br>KHYVGNVATLSWSELTFDEVVAPGAFPRLAALVRAANVEVDNDVYRSVVEWVAGIPDKRRVAFNM<br>NAFLGPDVVGSSWQGLSAHQTWDFGFGTSKAIRWPKPDLDGFVFYYPSRNTGDPDEGVEMVVCLE<br>DSAMQKLLKDPEWLNYAVAKGPRSCFDLDVAGA
>NpHCT2<br>MATVKVVESELVFPGEETPNHRLWLSNLDIMHALKLPTPLLYVYYPNGDSDFFNVAALKLGLSKALVP<br>YYPLAGRLNKGPDGRLEIDCNSKGALFVVAHSETCIDEFGDFLPSPEMRRLLTPSIPVDDPAVLLMVQV<br>TFFKGGEVILGLAWNHVVGDGVSLNSFMTSWCKMTQGITELGNPPIHDRTILRARSRPKVTFDHVGN<br>QLPTQIHSAQSPTTCSIFKLTKSQLAFLRKNNKFSTFDVVGAHVWRCVCIARGLGRDQEARLNFPIDA<br>RGHLRPPLPNSYFGNVVCRLVVVATVDDIISNPIDFTAHKIRGEIKKVNDEYVRSYIDHLEVSTKKKLML<br>TPAERNTTESTMLPKDSSNSSPSCEPESIAPQTQDNFLLTPSKMDFGVVSWLGFPFSGTDFGWGKPKI<br>LRRANISHGCYAYAMDSLVNDGEVLVPVLLETENMERFKKAFSEQLSMHSVERMEKPRSKY
>OsHCT3<br>MAVEIVKSSMVTAGEATPEHRIWLSNLDLLVARSHTPTVYVYRRTGPDSDAAFFSPDVLKAALSKVLV<br>PFYPLAGRLAQDSAGRPEISCTGEGVLFVTARSGATIDDLGDLAPSDELRRMLVPAADVAAASILAMF<br>QVTFFRCGGVCLGAAIHHTAADGLAALDFVNTWAAIARDVAGDGEAAAAAVQRPWLDRTLLRARS<br>PPAVRFDHAEYSRRRGGGSKLPFDSAILPMSKNQLNALKGAGAGAGKRLSTFTAVVAHVWRCACKA<br>RGLAVAGTEAATRLYMTADARTRLHPPLPRGYLGNAIFRASAVSKVSDIVAAGPLGAVAEKVSAATAR<br>LDDGYVRSLLDHLEQTAAAASGGAAGLRKGEWVMPESDLWVISWQGLPLYDADFGWGRPAFMGR<br>ACLQFSGLVYLVPGRDDGDGRLDVVVAMDPESLAKFKDVFYEELKC
>OsHCT4<br>MATVDVLTSEVVVPAGETPAGAVWLSNLDLAARRGYTPTVFFYRHNGEPGFFAADAMRDSLARALV<br>AFYPVAGRLGLDGDGRVQVDCTGEGVVFATARSGHYALDDLMGEFVPCDEMRDLFVPAAPAAASC<br>CPRGGALLLVQVTYLRCGGVVLGMALHHSIADGRSAAHFVETWASIARGAPAADAPVPPCFDHRLL<br>AARPARAVLYDHPEYKPEPAPPARAATASTYASAIITLTKQQVGALRAACAGASTFRAVVALVWQCA<br>CRARALPPEAETRLHSMIDTRQRLSPPLPPGYFGNAVIRTSTAATAGEVVSSPVGHAARRARAATSQG<br>EDYARSVVDYLEGVDAMNLPRSGVSRADLRAISWLGMSLADADFGWGSPAFMGPAIMYYSGFVYV<br>MNAPGKDGAVALALSLEPESMPEFRKVFADEVARLA

>NpHCT3
MVAIEVVKSELVFPSEETPKHRLWISNTDIMFATKLPTPMIYVYHPNGDPDFFNVEMLKAGLSKALVPF
YPLAGRLAKDRDGRLEVDCGRQGVLVVVARSDSGIDDFGEFLPSPELCRMLTPSIPSEDPDILFMTQVT
FFKGGEVILGTVWCHVIGDGLSAMNFISTWSRITRGVVSNLDIHPLHDRTLLRARSPPKVLFNHVGNQ
LPACAATTTGPSATSILPLTKSQLALLTTNNKISTFCAISAHVWKCVSIARGLARDQAVQLKFVVNVRR
RMKPPLPDDYFGNAVCRMVVVSTADDVLSNPIEVVGNKIRDEIRKVDEEYVRSFIDHFEMSPTKGAKP
PASSAKFSLTPTMADFAMASWIGMPIYDADFGWGKPEIVRIAAITDGQYAYIMEIPKGGGVLVLVSLEE
ENMERFKEGFYEGLNMHGTIRTD

Supplementary Table S2. Primers used in the study.

Primer name	Primer sequence (5'–3')
ZjHCT-qPCR-F	GAGATGCGGGACCTGTTCGT
ZjHCT-qPCR-R	TACAGCACCGTTGGCGTGG
ZjHCT-F	ATGGCTTCAAGGACGACAGTTA
ZjHCT-R	GAGAAGGAAAGCAAGCCAGAA
35SYFP-ZjHCT-F	GGGGACTCTTGACCATGGTAATGGCGACGGTGGAGGTGTT
335YFP-ZjHCT-R	ACACGCGTACTAGTCAGATCGCCACCTCCAGGCTCGCCA
UBI-ZjHCT-F	GTGTTACTTCTGCAGAGGATGGCGACGGTGGAGGTGTT
UBI-ZjHCT-R	TAATCCAGATCTACCATAGGCTACGCCACCTCCAGGCTC
ZjACT-F	GGTCCTCTTCCAGCCATCCTTC
ZjACT-R	GTGCAAGGGCAGTGATCTCCTTG
AsACT-F	GAGGTCCTTCCTGATATCCA
AsACT-R	CCTTTTCCAGCCATCTTTCA
ASCSE-RT-F1	CCACTCTGGCAGACCAAT
ASCSE-RT-R1	TAGTCCATCCCGAACACG
ASCAD5-RT-F1	TCAGGTATCGCTTCGTCATC
ASCAD5-RT-R1	CATCCATCTCCAGCACAGTTT
ASCAD6-RT-F1	TCATTCCCGCTTATCTTCG
ASCAD6-RT-R1	AGCCTGGACCCATTCCCT
ASCAD8B-RT-F1	AAGCAAACCAAGAAGGCAGAT
ASCAD8B-RT-R1	TGTCAGGACCGAAAGGAGC
ASCCR2-RT-F1	TTCTTCGGATCACATGGGTC
ASCCR2-RT-R1	TCGGTAAACCGTGTTCTTTCT
ASCCR1C-RT-F1	AAAGAAGAGCGGTAAAGGTG
ASCCR1C-RT-R1	AAGCTAGTACGCAAGACTGGTA
ASPAL-RT-F1	ACCAGGGAAAGCACATCG
ASPPAL-RT-R1	TTGGCATCGGCATAGCAG
AS4CL5-RT-F1	GGGTTATGGGATGACAGAGG$
AS4CL5-RT-R1	CCGGTCCACGATGAAGAT$
AS4CLL7-RT-F1	GGGATGTCATTTACCCTGCTA$
AS4CLL7-RT-R1	TGTTCTCCGTATGCCTTGC$

AS4CLL4-RT-F1	CTTTGCTTAGTGGGTTGTTG$
AS4CLL4-RT-R1	CTGCTGCTTATTGTGATGGT
ASPOD1-RT-F1	TCACCGAATCTCAAATACCA
ASP0D1-RT-R1	CAAGTGCTCGTCATGTCTCA
ASCOMT-RT-F1	GGTTTCCCGTTGAAGCAC
ASCOMT-RT-R1	GCACGAGCACCTTTGACT
ASCCOAOMT1-RT-F1	CAAGAACGGGAAGAGCAG
ASCCOAOMT1-RT-R1	CAGGGAAGTGACAGCAAA
ASCCOAOMT2-RT-F1	CTCGTAGTATTCCCGGTTCA
ASCCOAOMT2-RT-R1	CTTCTCCGTCCTGCTCAA
ASCSE-RT-F1	CCACTCTGGCAGACCAAT
ASCSE-RT-R1	TAGTCCATCCCGAACACG

`
